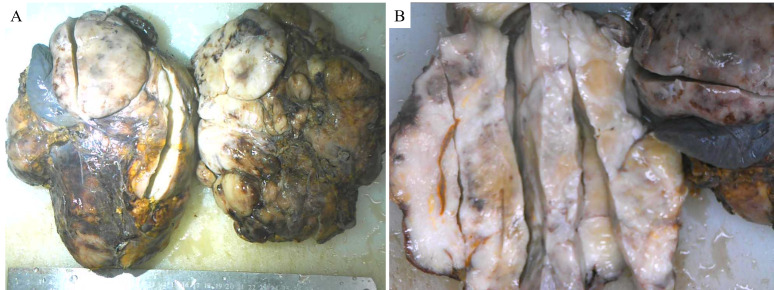# Recurrence of locally invasive retroperitoneal dedifferentiated liposarcoma shortly after surgery: A case report and literature review

**DOI:** 10.1097/MD.0000000000037604

**Published:** 2024-03-29

**Authors:** Xuelian Hu, Bo Han, Qin Yang, Qixuan Li, Dongkai Xiao, Xiaosong Xu

**Affiliations:** a Department of Nephrology, First Affiliated Hospital (Southwest Hospital), Army Medical University (Third Military Medical University), Chongqing, China; b Department of General Surgery, 63650 Military Hospital, Xinjiang, China; c Department of Medical Service, First Affiliated Hospital (Southwest Hospital), Army Medical University (Third Military Medical University), Chongqing, China.

**Keywords:** Case report, invasiveness, lessons learned, recurrence, retroperitoneal dedifferentiated liposarcoma

## Abstract

**Rationale:**

Retroperitoneal dedifferentiated liposarcoma (RPDDL) is an uncommon malignancy, which often remains undetected for many years due to having adequate space in the retroperitoneal cavity and lacking clinical manifestations in the early stage of the disease. Surgical procedure is usually used as the first choice for treatment. However, it is prone to local recurrence after the operation, resulting in an unfavorable prognosis. Our aim is to draw useful lessons from the new case and provide some experience for management of the disease.

**Patient concerns:**

We describe a 55-year-old male patient who was admitted for a 3-week history of persistent dull ache of the left waist. A large mass of the left upper abdomen was palpated in physical examination. Moreover, the imaging examination revealed that the diameter of the mass was about 21 cm, and some adjacent vital organs were invaded, which brought great challenges to complete surgical resection.

**Diagnosis:**

The postoperative pathological results confirmed that the mass was RPDDL with invasion of the surrounding vital structures including pancreas, spleen, left adrenal gland, left kidney, and vasculature with tumor emboli.

**Interventions:**

Surgical resection of the mass was performed by our multidisciplinary team. The patient received chemotherapy 1 month after surgery.

**Outcomes:**

The effect of chemotherapy seemed to be unsatisfactory. Local multifocal recurrence of the tumor was considered about 2 months after surgery. Finally, he gave up any treatments and died of the disease.

**Lessons:**

Regular physical examination and ultrasound screening may detect the disease as early as possible, especially for high-risk group aged 60 to 70, which should be popularized. Incomplete resection, vascular invasion, and interruption of postoperative treatment may lead to an unfavorable prognosis. Therefore, we think that patients with the disease may benefit from complete surgical resection and uninterrupted adjuvant therapy.

## 1. Introduction

Soft tissue sarcoma (STS) is a relatively rare malignant tumor, accounting for only 1% of all adult tumors.^[1,2]^ As one of the most common types of STS, retroperitoneal liposarcoma (RPLS) makes up nearly 30% of retroperitoneal sarcoma (RPS).^[3]^ Although RPLS can occur in any age group, it generally affects people between the ages of 60 and 70.^[4,5]^ To date, no specific etiological association of RPLS has been established. Due to the enough space in the retroperitoneal cavity, patients with RPLS usually have a long course without any discomforts in the early stage. The nonspecific symptoms, including abdominal distention or pain, nausea, vomiting and so on, mostly do not appear until tumors are large enough to compress or invade the surrounding vital structures.^[1,4,6]^ According to the definition of the World Health Organization, RPLS can be classified into 4 major subtypes: well-differentiated (WDL), dedifferentiated (DDL), myxoid, and pleomorphic.^[7]^ Among them, retroperitoneal dedifferentiated liposarcoma (RPDDL) represents approximately one-third, about 90% arising de novo and 10% following recurrences of retroperitoneal well-differentiated liposarcoma (RPWDL).^[5,8]^ Moreover, surgical resection remains the most efficacious treatment modality for RPDDL, while the roles of radiotherapy and chemotherapy have been controversial.^[1,2,4,6,9–11]^ Compared to RPWDL, RPDDL frequently has obvious malignant biological behavior with a tendency to recur following surgical resection, which can often lead to a poor prognosis.^[12,13]^ Therefore, it is of great clinical practice value to summarize the novel cases and review the literature to optimize diagnosis and treatment plan. We hereby elucidate a case of a middle-aged man presenting with a large RPDDL invading the surrounding vital structures.

## 2. Case presentation

### 2.1. Patient and treatment

**Figure 1.**
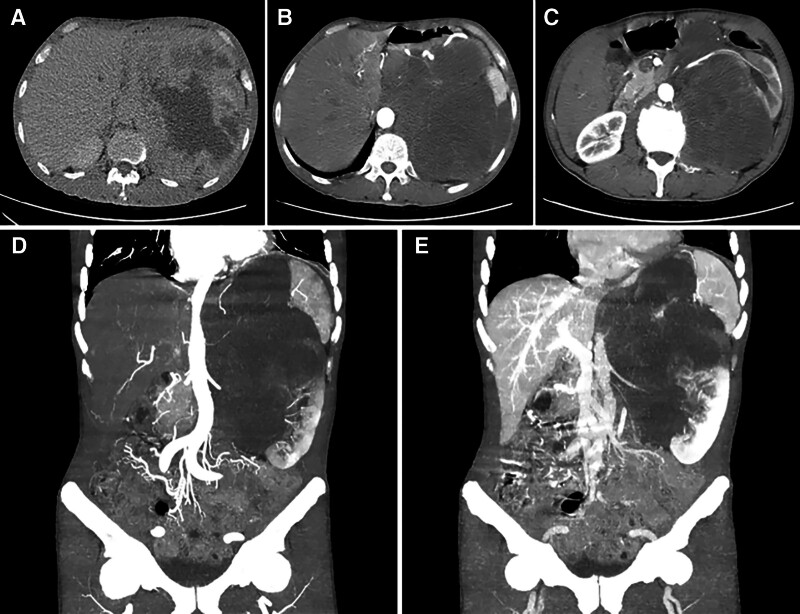
CT scan of the retroperitoneal tumor. (A) There was extensive necrosis in the tumor. (B) The abdominal aorta was compressed to the right of the spine. (C) The left renal artery passed through the tumor. (D) The tumor pushed bowels into the right abdomen and pelvis. (E) The tumor significantly invaded the left kidney. The splenic vessels were wrapped in the tumor.

**Figure 2.**
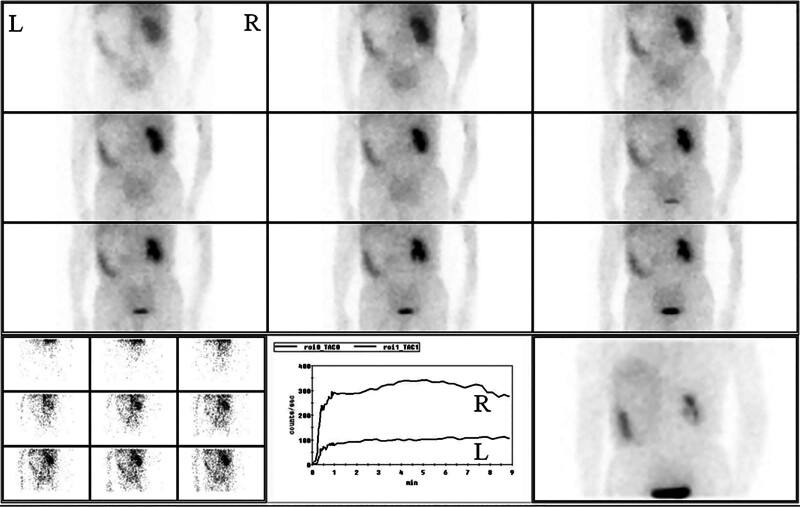
ECT examination of the bilateral kidneys. The left kidney exhibited poor imaging and severe decline in function. (L) Left side. (R) Right side.

This 55-year-old male patient had a 3-week history of persistent dull ache of the left waist. He complained that the pain was evident at night and could be slightly relieved by appropriate movement of his waist. Meanwhile, his mental state and sleep quality were poor, and the food intake decreased by about 50%. There was no obvious abdominal pain, nausea, vomiting, and others during the course of disease. No history of abdominal special disease, surgery, or trauma had been confirmed. Ultrasound examination at the local hospital indicated that the left upper quadrant of the abdomen had a large mass. It was thought to be possibly originated from the left kidney. To obtain definite diagnosis and active treatment, he was admitted to our department. Physical examination on admission found that a mild distension of the left upper abdomen was carefully observed, a large mass with solid texture and poor range of motion was palpated, and an obvious percussive pain in the left kidney area was discovered. No abnormality was detected in blood routine, urinalysis, thyroid function, liver function, kidney function, and blood clotting index. Ultrasound examination revealed a large hypoechoic lesion with an obscure boundary in the left upper quadrant of the abdomen. Abdominal computed tomography (CT) scan indicated that the diameter of the heterogeneous soft tissue mass with low density was approximately 21 cm, some surrounding vital structures involving left kidney, spleen, pancreas, stomach, bowels, and vessels were compressed and displaced, the line-like enhancement was observed on contrast-enhanced scan, and the blood vessels of the left kidney and partial abdominal aorta were enveloped by the mass (Fig. 1). Emission computed tomography demonstrated that the solid lesion of the left upper abdomen was slightly enhanced, the left kidney was displaced obviously, the glomerular filtration function of the left kidney was decreased by about 52% (Fig. 2), and no definite abnormal metabolic signs were found in the bones of the whole body (Supplementary Fig. 1, http://links.lww.com/MD/L1000).

**Figure 3.**
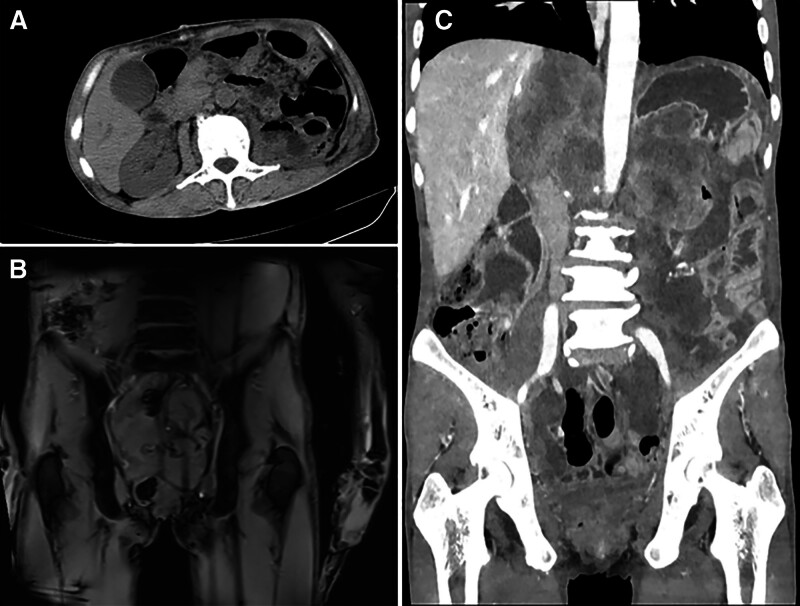
Abdominal imaging examination after the operation. (A) No obvious residual tumor was discovered in the retroperitoneal cavity. (B) MRI revealed multiple lesions in the pelvis. (C) CT showed multiple lesions in the abdomen.

The patient underwent extensive surgical resection after adequate preoperative preparation. During the operation, it was discovered that the retroperitoneal tumor closely adhered to the left kidney, left adrenal gland, spleen, gastric body, and descending colon. Furthermore, the body and tail of pancreas and the left renal artery and vein were wrapped in the tumor. Therefore, our multidisciplinary team, consisting of nephrologist, pancreatic surgeon, general surgeon, and anesthesiologist, performed complete resection of the retroperitoneal tumor, radical resection of the left kidney and adrenal gland, distal pancreatectomy, splenectomy, and lysis of intestinal adhesions. The left diaphragm was damaged during intraoperative separation and then repaired with continuous suture. Owing to the abdominal aorta tightly adherent to the tumor, an about 0.3 cm in diameter vascular injury was occurred despite all our efforts to avoid it. No definite active bleeding was observed after repair with 4-0 prolene suture. The total blood loss was about 5500 mL. Considering that the patient suffered from hemorrhagic shock, 3150 mL of isotonic crystalloid solution, 18 U of red blood cells suspension, and 2130 mL of fresh frozen plasma were transfused for treatment during the surgery. In the meantime, norepinephrine was continuously pumped into the blood to maintain systolic pressure at 100 to 120 mm Hg. The highest value of blood lactic acid was 2.5 mmol/L, monitored by intraoperative blood gas analysis. The patient was transferred to intensive care unit because of the unstable blood circulation and the high-risk of complications such as acute respiratory distress syndrome, pancreatic leakage, severe abdominal bleeding or infection, and multiple organ dysfunction syndrome. In the aspect of treatment, he was given ventilator-assisted breathing, intensive care, active anti-infection, nutritional support, inhibition of pancreatic enzyme, protection management of important organs, etc. On the second day after the operation, his spontaneous respiration was steady without relying on the ventilator, and blood pressure was maintained at normal level without using any vasoactive drugs. On the third day, he was moved to a general ward of our department. Subsequently, the hemoglobin was monitored continuously, decreasing from 128 to 67 g/L. After transfusing 3 U of red blood cells suspension and 600 mL of fresh frozen plasma, and strengthening enteral and parenteral nutrition, it gradually recovered to more than 100 g/L. Abdominal CT reexamination showed that there was no obvious residual tumor in the left retroperitoneum (Fig. 3A). He was discharged successfully on the 18th day after surgery. One month later, he returned to our hospital and received the TA (paclitaxel liposome and epirubicin hydrochloride) chemotherapy regimen once. Unfortunately, about 2 months later, local recurrence of the tumor was considered because multiple pelvic and abdominal lesions were discovered by Magnetic Resonance Imaging and CT (Fig. 3B, C). In the end, he was forced to give up any treatments due to the high cost of the subsequent medical care and died of the disease half a year later.

### 2.2. Pathology

**Figure 4.**
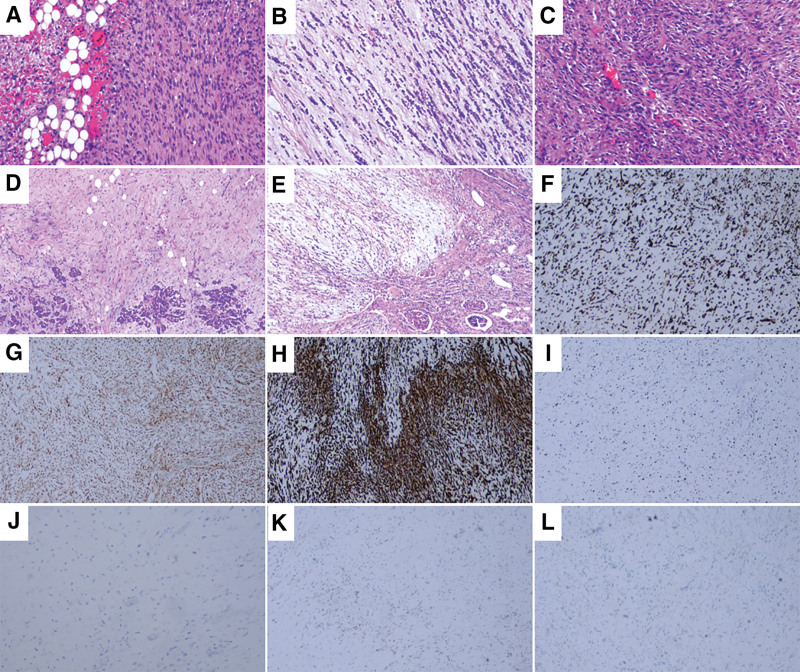
Histochemical staining of the resected tumor. (A–E) Hematoxylin–eosin staining. (A) There were well-differentiated and dedifferentiated areas in the tumor (×100). (B) There was myxofibrosarcoma area in the tumor (×100). (C) There were atypical spindle cells in the tumor (×100). (D) The tumor invaded the pancreatic tissue (×40). (E) The tumor invaded the left renal tissue (×40). (F–L) Immunohistochemical staining. (F) The CDK4 staining of the tumor cells was positive (×40). (G) The MDM2 staining of the tumor cells was positive (×40). (H) The P16 staining of the tumor cells was positive (×40). (I) The number of Ki67 positive cells was about 30% (×40). (J) The CK staining of the tumor cells was negative (×100). (K) The Rb staining of the tumor cells was negative (×40). (L) The SOX-10 staining of the tumor cells was negative (×40).

On gross examination, the retroperitoneal mass approximately measured 25 × 18 × 16 cm with grayish-white or grayish-yellow longitudinal sections (Supplementary Fig. 2, http://links.lww.com/MD/M2). It adhered to the surrounding tissues and organs, which were not clearly demarcated from the surrounding structures. The sizes of attached pancreatic, splenic, and renal tissue were about 6 × 5 × 1.2 cm, 8 × 5 × 4 cm, and 11 × 7.5 × 2.5 cm, respectively. The normal internal structure of the left kidney was severely damaged. Microscopically, the results of the immunohistochemical staining revealed that the tumor cells were positive for cyclin-dependent kinase 4 (CDK4) (Fig. 4F), mouse double minute 2 homolog (MDM2) (Fig. 4G) and P16 (Fig. 4H), but negative for cytokeratin (CK) (Fig. 4J), retinoblastoma protein (Rb) (Fig. 4K), and SRY-box transcription factor 10 (SOX-10) (Fig. 4L). The number of Ki67 positive cells was about 30% (Fig. 4I). Furthermore, MDM2 gene amplification was verified by fluorescence in situ hybridization. Consequently, the pathological findings supported the diagnosis of RPDDL invading the surrounding vital structures including pancreas, spleen, left adrenal gland, left kidney, and vasculature with tumor emboli.

## 3. Discussion

RPDDL seriously affecting the health of adults has been generally defined as a nonlipogenic neoplasm originated from the retroperitoneum with WDL juxtaposed to an intermediate-grade to high-grade myxofibrosarcoma or a high-grade malignant fibrous histiocyoma-like pleomorphic sarcoma.^[14]^ Although the specific pathogenesis of the disease remains unknown, it is closely associated with genetic abnormalities mainly involving chromosome 12q13-15, with amplification of the oncogenes including MDM2 (100%) and CDK4 (90%).^[15]^ Genetically, DDL is similar to WDL but has more genetic alterations, particularly 1p32 and 6q23 co-amplifications. Moreover, clinically, DDL has a more aggressive behavior than WDL that can transform into DDL but does not metastasize.^[16]^

RPDDL is a deep, painless, and growing malignant tumor. Owing to the large space in the retroperitoneal area, most patients can have no discomfort during the course of disease. Some symptoms usually occur when it is large enough to compress or invade the surrounding vital structures. Typically, it may reach a large size at diagnosis.^[1,4,6]^ Thus, the early diagnosis of RPDDL is an enormous challenge. However, in our opinion, annual health examinations, including physical examination and ultrasound screening, may discover the disease as early as possible, especially for high-risk group aged 60 to 70. On the one hand, the incidental discovery of an asymptomatic abdominal mass is the most common manifestation through a physical examination.^[17]^ On the other hand, the abdominal ultrasound screening is the quickest, simplest, and most useful method for locating the tumor and measuring the size.^[11]^ In addition, contrast-enhanced CT and MRI also are important imaging techniques to evaluate the retroperitoneal mass, which are helpful to determine its location, origin, size, and relationship with the adjacent tissues and organs.^[11]^ Generally, RPDDL manifests as a heterogeneous fatty mass with solid nodules and internal septations.^[1,6]^ But a few cases reported that RPDDL appeared as the huge cystic degeneration or contained the osteosarcomatous components with extensive calcification.^[14,18,19]^ Furthermore, positron emission tomography-computed tomography (PET-CT) can be performed to estimate tumor grade and to obtain information on metastases.^[11]^ Because of its high cost, only a small number of patients, to our knowledge, agree to use it in our country. When the imaging findings are unclear, percutaneous core needle biopsy can establish a relatively clear diagnosis and provide a basis for appropriate preoperative treatment, but its overall accuracy is not high for DDL, and there is a risk of tumor cells implantation and dissemination, so its necessity has not been concluded.^[1]^

Complete surgical resection is the most effective treatment for RPDDL.^[6]^ However, it is sometimes difficult to achieve complete resection in clinical practice, because the tumor is usually large and involves the adjacent vital organs.^[1,11]^ It had been reported that approximately 50% of cases required multiple organs excision to reach complete resection.^[11]^ Nevertheless, unlimited extensive resection of the uninvolved structures not only does not improve survival benefit but also increases postoperative mortality.^[1]^ Therefore, sufficient preoperative evaluation, careful intraoperative exploration, and accurate resection are especially critical. At present, there is a lack of persuasive evidence for adjuvant treatment of RPDDL. Despite having many controversies about the efficacy of radiotherapy and chemotherapy, they may play a role in cases of unresectable tumors, incomplete excisions with positive margins and metastatic tumors.^[4]^ It had been reported that preoperative radiotherapy improved the negative rate of surgical margins and reduced the postoperative local recurrence rate, but no survival advantage was established compared to surgery alone.^[1]^ Moreover, some case reports had demonstrated the significant effectiveness of neoadjuvant chemotherapy or chemotherapy in RPDDL.^[10,20]^ In our case, the patient underwent the TA chemotherapy regimen after surgery but did not seem to get benefit from it.

As a matter of fact, surgical resection is still the first selection of local recurrent RPDDL. However, due to the invasive growth of the tumor and the multifocal recurrence shortly after operation, the feasibility of reoperation is not high in this case. Targeted therapy is another choice for the treatment of recurrent RPDDL. Anlotinib, a novel tyrosine kinase inhibitor, had been shown to have broad-spectrum antitumor activity against metastatic STS that progressed after the use of anthracycline-based chemotherapy.^[21]^ Another study revealed that anlotinib was an effective treatment option for unresectable local recurrence or metastatic WDL/DDL.^[22]^ More than 90% of WDL/DDL have CDK4 amplification.^[23]^ Palbociclib, a potent inhibitor of CDK4, could improve progression-free survival of patients with advanced WDL/DDL and achieve long-term stabilization of disease.^[24]^ A recent article also reported relatively favorable clinical control of palbociclib for RPDDL patient with lung metastasis after surgery.^[25]^ Unfortunately, the patient in our case did not receive any targeted drugs due to personal financial situation.

The 5-year overall survival rates have been reported to range from 44% to 53% in RPDDL. Local recurrence is a common cause of death, occurring in 40% to 80% of the patients. Distant metastases have been observed in 15% to 20% of the cases, and the most common metastatic organ is the lung. The important factors affecting survival and prognosis include completeness of the resection, status of the margin, grade of the histology, and so on.^[13]^ Luo et al^[5]^ revealed that complete tumor resection, tumor integrity, and intermediate-grade histology were independently predictors of better overall survival (OS), and tumor integrity, intermediate-grade histology, and unifocal disease independently predicted favorable Progression-Free Survival. Dehner et al^[26]^ discovered that local recurrence-free survival of patients with the dedifferentiated component at the margin (positive margin) was significantly shorter than those with the negative margin. They recommended that the presence or absence of the positive margin needed to be reported to add meaningful prognostic information.

In our case, the patient’s prognosis was unfavorable, and local recurrence occurred shortly after the operation. As far as we are concerned, the reasons may be as follows: since the actual status of the surgical margin was not obtained, the reliability of complete resection was questionable, and the unrecognizable residual tumor might be left during surgery; the pathological results showed that the large tumor had invaded the vasculature, so the risks of local recurrence and distant metastasis were greatly increased; The patient was only treated with chemotherapy in the early stage after the operation, but the effect did not respond well; the patient did not receive targeted therapy or radiotherapy and gave up the subsequent treatment after recurrence.

To sum up, RPDDL is an uncommon and asymptomatic malignancy located in the retroperitoneum. It is pretty difficult to be discovered in the early stage of the disease. Generally speaking, the size of the tumor is often large, and some surrounding vital structures are invaded at the time of the discovery. Hence, the annual health examinations are quite important in early detection. Surgical resection is currently the only effective method supported by a mountain of evidence, but the probability of local recurrence is still high, so active adjuvant therapy and regular follow-up are absolutely indispensable. Incomplete resection, vascular invasion, and interruption of postoperative treatment may lead to an unfavorable prognosis. Therefore, popularization of annual health examinations, complete surgical resection, and uninterrupted adjuvant therapy are especially significant for management of the disease.

## Acknowledgments

We would like to thank the patient for participating in this study.

## Author contributions

**Conceptualization:** Xiaosong Xu.

**Data curation:** Qixuan Li.

**Formal analysis:** Bo Han, Dongkai Xiao.

**Investigation:** Xuelian Hu.

**Methodology:** Bo Han.

**Project administration:** Qin Yang, Xiaosong Xu.

**Resources:** Qin Yang, Qixuan Li, Dongkai Xiao.

**Software:** Bo Han.

**Supervision:** Qin Yang, Dongkai Xiao, Xiaosong Xu.

**Validation:** Qin Yang, Qixuan Li.

**Visualization:** Xuelian Hu.

**Writing—original draft:** Xuelian Hu, Bo Han.

**Writing—review & editing:** Xiaosong Xu.

## Supplementary Material

**Figure:**
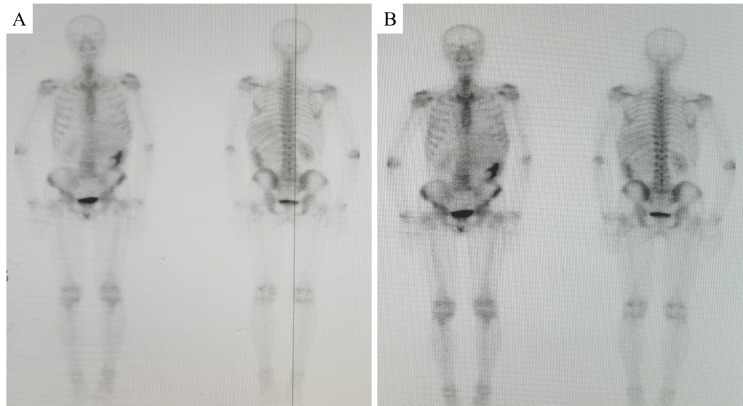


**Figure:**